# MAXWELL A. POLLACK AWARD FOR CONTRIBUTIONS TO HEALTH AGING PRESENTATION AND LECTURE

**DOI:** 10.1093/geroni/igac059.385

**Published:** 2022-12-20

**Authors:** Philip Rozario

## Abstract

The Maxwell A. Pollack Award for Contributions to Health Aging Lecture will feature an address by the 2021 Pollack Award recipient Namkee G. Choi, PhD, FGSA, of the University of Texas at Austin. This session will also include the presentation of the 2022 Maxwell A. Pollack Award to recipient Nancy Morrow-Howell, MSW, PhD, FGSA, of Washington University in St. Louis. The Maxwell A. Pollack Award for Contributions to Healthy Aging Award recognizes instances of practice informed by research and analysis, research that has directly improved policy or practice, and distinction in bridging the worlds of research and practice.

